# A Systematic Review and Meta-Analysis of Randomized Clinical Trials of the Efficacy of Mesalazine in Irritable Bowel Syndrome

**DOI:** 10.7759/cureus.110300

**Published:** 2026-06-05

**Authors:** Rogelio Santos, Christian Navarro

**Affiliations:** 1 Department of Internal Medicine, Hospital General Regional No. 1, Instituto Mexicano del Seguro Social, Chihuahua, MEX; 2 Department of Gastroenterology, Hospital Ángeles Chihuahua, Chihuahua, MEX

**Keywords:** 5-asa, ibs, mesalazine, meta-analysis, randomized controlled trials

## Abstract

Mesalazine (5-aminosalicylic acid, 5-ASA) has been proposed as a therapeutic option for irritable bowel syndrome (IBS) due to its anti-inflammatory properties, but its efficacy remains uncertain. This systematic review and meta-analysis evaluated randomized controlled trials comparing mesalazine versus placebo in adult patients with IBS. A systematic search of PubMed, ScienceDirect, and Google Scholar was conducted up to March 23, 2026. Only the first 100 Google Scholar results sorted by relevance were screened. Risk ratios (RRs) and standardized mean differences (SMDs) were pooled using a random-effects model, and risk of bias was assessed using the Cochrane RoB 2 tool. Seven randomized controlled trials involving 569 participants were included. Mesalazine did not significantly improve global IBS symptom response compared with placebo (RR 1.11, 95% CI 0.93-1.32; I² = 10%). No significant effect was observed for abdominal pain (SMD -0.10, 95% CI -0.82 to 0.61; I² = 83%). However, mesalazine significantly improved global IBS symptom scores (SMD -0.39, 95% CI -0.64 to -0.15; I² = 16%). The overall risk of bias was low to moderate across studies. In conclusion, mesalazine does not significantly improve global IBS response or abdominal pain, but it may modestly reduce overall symptom burden. Further high-quality randomized controlled trials are needed to identify whether selected IBS phenotypes with mucosal immune activation may benefit from mesalazine.

## Introduction and background

Irritable bowel syndrome (IBS) is a highly prevalent disorder of gut-brain interaction characterized by recurrent abdominal pain associated with changes in bowel habits, affecting approximately 5-10% of the global population and significantly impairing quality of life and healthcare utilization [1-3]. Current gastroenterology guidelines emphasize that IBS should be diagnosed using a positive symptom-based approach, while also excluding alarm features and alternative diagnoses when clinically indicated [4,5]. Unlike inflammatory bowel disease (IBD), IBS is not defined by overt mucosal ulceration, structural intestinal damage, or chronic relapsing inflammation; however, growing evidence suggests that a subset of patients may exhibit low-grade immune activation and mucosal inflammation [6-8].

IBS is a heterogeneous condition with multifactorial pathophysiology, including altered gut-brain signaling, visceral hypersensitivity, abnormal motility, microbiota dysbiosis, increased intestinal permeability, and immune activation [6-8]. In the past decade, increasing attention has been given to the role of low-grade mucosal inflammation in IBS. Studies have reported increased infiltration of immune cells, including mast cells and lymphocytes, as well as elevated levels of pro-inflammatory cytokines in selected IBS populations [9-11]. This inflammatory component appears to be particularly relevant in post-infectious IBS, where persistent immune activation following an acute gastrointestinal infection may contribute to symptom persistence [12-14].

Current IBS guidelines recommend individualized, symptom-directed management, including dietary modification, gut-brain behavioral therapies, antispasmodics, neuromodulators, antidiarrheal agents, secretagogues, and selected microbiota-directed therapies depending on the predominant bowel habit and clinical phenotype [4,5]. However, anti-inflammatory therapies such as mesalazine are not routinely recommended in standard IBS management because the available evidence remains limited and inconsistent. This gap is clinically relevant because a subset of IBS patients may have immune activation or post-infectious disease mechanisms that could theoretically respond to targeted intestinal anti-inflammatory therapy.

Mesalazine, also known as 5-aminosalicylic acid, is an established anti-inflammatory therapy widely used in IBD. Its proposed mechanisms include inhibition of cyclooxygenase and lipoxygenase pathways, reduction of pro-inflammatory mediators, modulation of mucosal immune responses, and local anti-inflammatory effects within the intestinal mucosa [15,16]. Given its favorable safety profile and targeted intestinal activity, mesalazine has been investigated as a potential therapeutic option for IBS, particularly in patients with suspected immune activation or post-infectious disease.

However, randomized clinical trials evaluating mesalazine in IBS have yielded conflicting results. Some studies have reported modest improvements in global IBS symptoms, abdominal pain, or inflammatory biomarkers, whereas others have failed to demonstrate significant benefit compared with placebo [17-19]. These inconsistencies may be explained by differences in patient selection, IBS subtype, post-infectious status, treatment dose, treatment duration, and outcome definitions.

Although previous evidence has explored the potential role of anti-inflammatory therapy in IBS, the efficacy of mesalazine remains uncertain and has not been clearly established across randomized placebo-controlled trials. Therefore, we conducted a systematic review and meta-analysis of randomized controlled trials (RCTs) to synthesize the available evidence and evaluate the efficacy of mesalazine for global symptom response, abdominal pain, and overall IBS symptom improvement. By pooling trial-level data, this study aims to clarify whether mesalazine provides a clinically meaningful benefit in IBS and to address inconsistencies among previous randomized studies.

## Review

Methods

Study Design and Search Strategy

A systematic literature search was conducted and reported in accordance with the Preferred Reporting Items for Systematic Reviews and Meta-Analyses (PRISMA 2020) guidelines [18]. The review protocol was prospectively registered in PROSPERO, the International Prospective Register of Systematic Reviews (registration number: CRD42026315173).

Relevant studies were identified through comprehensive electronic database searches in PubMed, ScienceDirect, and Google Scholar from inception to March 23, 2026. The main search strategy combined terms related to the intervention and condition as follows: (mesalazine OR mesalamine OR 5-ASA) AND (irritable bowel syndrome OR IBS) AND (randomized)

Database-specific search strategies are provided in Appendix 1 (see Table 3) to improve transparency and reproducibility. The initial search was conducted without language restrictions.

For PubMed, the search strategy was adapted using free-text terms for mesalazine, mesalamine, 5-ASA, irritable bowel syndrome, IBS, and randomized trials. For ScienceDirect, the same core search terms were used, and filters were applied to include only original research articles; records were then screened based on title and abstract. For Google Scholar, the first 100 records, sorted by relevance, were screened because the database retrieves a very large number of records, with relevance decreasing after the initial pages. This approach was used as a pragmatic supplementary search strategy to identify additional gray literature and potentially missed studies while maintaining feasibility and reproducibility.

Additionally, manual screening of the reference lists of included studies was performed to identify any additional relevant randomized trials. Although additional databases such as EMBASE were not available, efforts were made to minimize selection bias through manual reference screening.

After removal of duplicated records, titles and abstracts were screened to identify potentially relevant studies, followed by full-text assessment against predefined eligibility criteria. Studies were considered eligible if they were randomized clinical trials involving adult patients diagnosed with irritable bowel syndrome. Trials had to compare mesalazine, mesalamine, or 5-aminosalicylic acid with placebo or another control intervention and report at least one clinically relevant outcome, such as global symptom improvement, global IBS symptom scores, abdominal pain, or adverse events.

Because this systematic review and meta-analysis used previously published data, ethical approval and informed consent were not required.

Study Selection

Study selection was conducted independently by two reviewers, and any discrepancies were resolved through discussion and collective decision.

A total of 540 records were identified by searching electronic databases. After the removal of five duplicate records, 535 records were screened based on title and abstract. Of these, 515 records were excluded.

A total of 20 reports were sought for full-text retrieval, of which two were not retrieved. Therefore, 18 full-text articles were evaluated according to predefined criteria. After full-text evaluation, 11 reports were excluded: six because they were not randomized controlled trials and five because they did not provide sufficient data for quantitative synthesis.

Ultimately, seven randomized controlled trials were included in the qualitative and quantitative synthesis.

Eligibility Criteria

We included randomized controlled trials that evaluated adult patients with irritable bowel syndrome and compared mesalazine with a placebo. To be eligible for quantitative synthesis, studies had to report extractable outcome data suitable for effect size calculation.

We excluded non-randomized investigations, observational studies, reviews, editorials, and case reports. Studies were also excluded when they did not evaluate patients with irritable bowel syndrome or when the available data were insufficient for extraction and quantitative synthesis.

Data Extraction

Two investigators independently extracted data using a standardized form. The collected information included study-level characteristics, including author, publication year, and study population; sample size; intervention and comparator details; outcomes of interest; and numerical data required for meta-analysis, such as means, standard deviations, and event counts.

When necessary, standard deviations were derived from reported data according to Cochrane recommendations [20]. Disagreements between reviewers during study selection and data extraction were resolved through discussion and consensus. If consensus could not be reached, a third reviewer was consulted.

Risk of Bias Assessment

We evaluated the risk of bias of the included studies using the Cochrane Risk of Bias 2 (RoB 2) tool [21]. The assessment included five domains: bias arising from the randomization process, deviations from intended interventions, missing outcome data, measurement of the outcome, and selection of the reported result.

Disagreements in risk of bias judgments were resolved by discussion and consensus, with consultation of a third reviewer when necessary.

Statistical Analysis

Meta-analysis was performed using Review Manager (RevMan version 5.4; Cochrane Collaboration, London, England, UK). For dichotomous outcomes, pooled effect estimates were expressed as risk ratios (RR) with 95% confidence intervals (CI). Continuous outcomes were analyzed using standardized mean differences (SMD) with 95% CI, as different studies used varying scales to measure symptom severity.

To account for expected clinical and methodological heterogeneity across studies, a random-effects model was employed. Heterogeneity was quantified using the I² statistic, with values exceeding 50% regarded as evidence of substantial heterogeneity. Sensitivity analyses were prespecified to examine the stability of the results where applicable, but they were not performed owing to the limited number of studies included.

Subgroup analyses and meta-regression were not performed due to the limited number of included studies and inconsistent reporting of IBS subtype, post-infectious status, and inflammatory biomarkers across trials.

Publication bias was not assessed using funnel plots because fewer than 10 studies contributed to each pooled outcome, making visual assessment of funnel plot asymmetry unreliable. Statistical significance was defined as a two-sided p-value <0.05.

Results

Search Results and Study Selection

The literature search identified a total of 540 records through database searching, including eight records from PubMed, 432 from ScienceDirect, and 100 from Google Scholar. After the removal of five duplicate records, 535 records were screened based on title and abstract. Of these, 515 records were excluded because they were not relevant to the review question or did not meet the predefined inclusion criteria.

**Figure 1:**
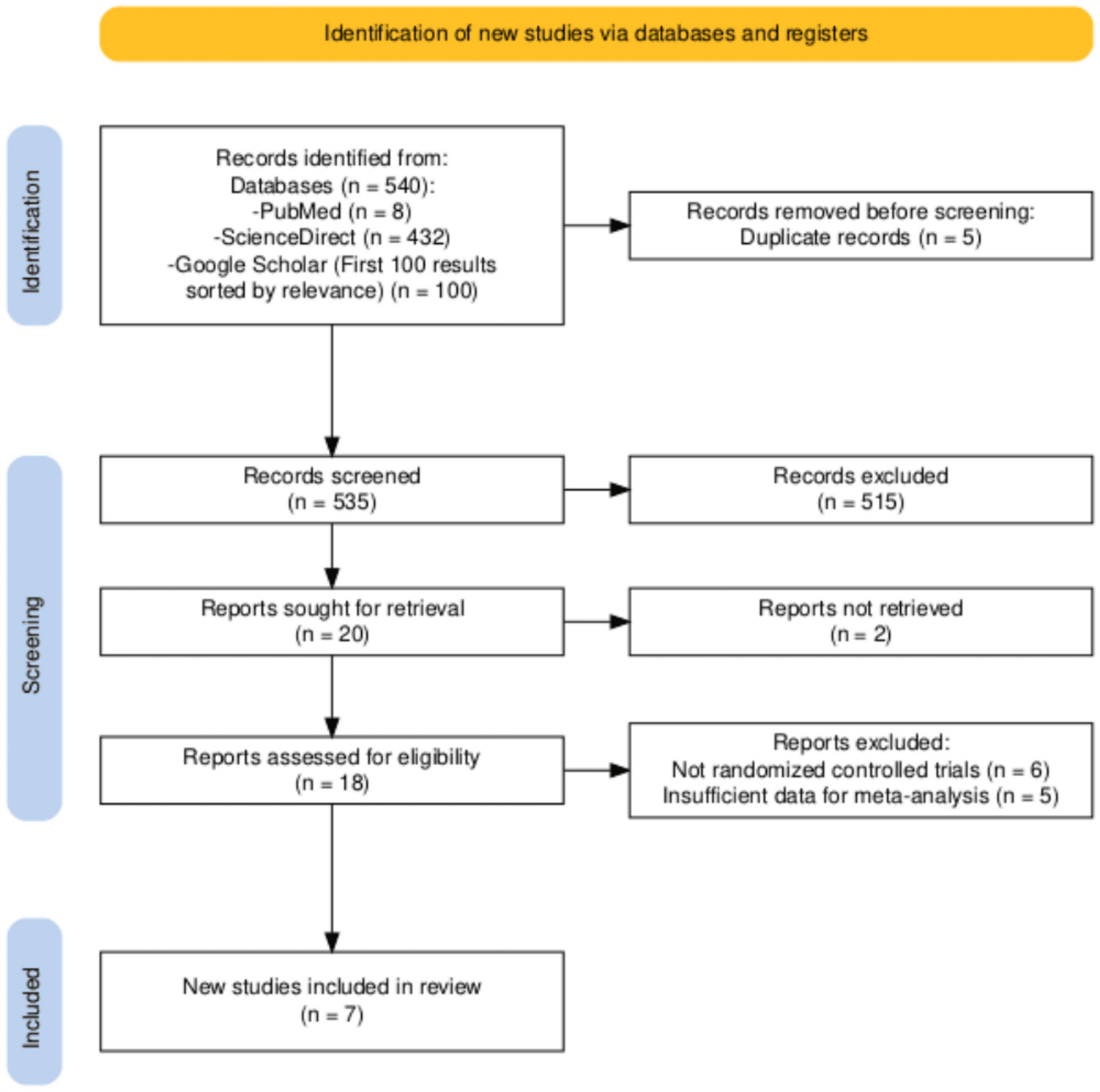
PRISMA 2020 flow diagram of study selection. PRISMA: Preferred Reporting Items for Systematic Reviews and Meta-Analyses. Flow diagram constructed according to PRISMA 2020 guidelines [18].

A total of 20 reports were sought for full-text retrieval. Of these, two reports were not retrieved, leaving 18 full-text articles assessed for eligibility. After full-text evaluation, 11 reports were excluded: six because they were not randomized controlled trials and five because they did not provide sufficient data for quantitative synthesis. Ultimately, seven randomized controlled trials (RCTs) were included in the qualitative and quantitative synthesis (Figure 1).

Study Characteristics

The final analysis included seven randomized controlled trials evaluating mesalazine versus placebo in patients with irritable bowel syndrome [22-28]​​​​​​. The included studies were conducted in diverse geographic settings, including the United States, Europe, and Asia. They included heterogeneous IBS populations such as mixed IBS, diarrhea-predominant IBS (IBS-D), and post-infectious IBS. Treatment duration ranged from 8 to 12 weeks.

Overall, the included trials varied in sample size, study design, and outcome definitions, reflecting the clinical heterogeneity of IBS. The main characteristics of the included randomized controlled trials are summarized in Table 1.

Among the included studies, four reported dichotomous data for global IBS symptom response, three reported continuous data for abdominal pain, and three reported continuous data for global IBS symptom score. Sample sizes were generally modest, ranging from 49 to 181 participants per study, which should be taken into account when interpreting pooled estimates.

Overall, the included trials demonstrated acceptable methodological quality. Most studies were judged to have a low risk of bias, although some concerns were identified in specific domains, particularly the randomization process and selective reporting.

**Table 1:** Characteristics of the included randomized controlled trials evaluating mesalazine versus placebo in patients with irritable bowel syndrome. This table summarizes the key characteristics of the seven studies included in the quantitative synthesis (meta-analysis). Data are presented as reported in the original studies [22-28]​​​​​​. IBS: irritable bowel syndrome; IBS-D: diarrhea-predominant IBS; M/P: mesalazine/placebo.

Study	Year	Country	Population (IBS type)	Sample size (M/P)	Intervention	Duration	Primary outcome	Key findings
Barbara et al. [22]	2016	Italy	IBS (mixed)	86/86	Mesalazine vs placebo	12 weeks	Global IBS response	No significant difference
Lam et al. [23]	2016	UK	IBS-D	57/58	Mesalazine vs placebo	12 weeks	Symptom severity	No significant effect
Castro Tejera et al. [24]	2022	Spain	IBS	91/90	Mesalazine vs placebo	12 weeks	Symptom score	Trend toward improvement
Aron et al. [25]	2012	USA	IBS (mixed)	51/50	Mesalazine vs placebo	12 weeks	Global IBS response	No significant improvement
Ghadir et al. [26]	2017	Iran	IBS	29/20	Mesalazine vs placebo	8 weeks	Abdominal pain	No significant improvement
Tuteja et al. [27]	2024	USA	Post-infectious IBS	28/26	Mesalazine vs placebo	8 weeks	Global symptoms	Significant improvement
Hossein et al. [28]	2025	Iran	IBS	45/45	Mesalazine vs placebo	12 weeks	Symptom severity	Mixed results

Quality Assessment

We evaluated the risk of bias of the included studies using the Cochrane Risk of Bias 2 (RoB 2) tool. The assessment considered five domains: bias arising from the randomization process, deviations from intended interventions, missing outcome data, outcome measurement, and selection of the reported results. Overall judgments were assigned as low risk of bias, some concerns, or high risk of bias according to RoB 2 criteria.

**Figure 2:**
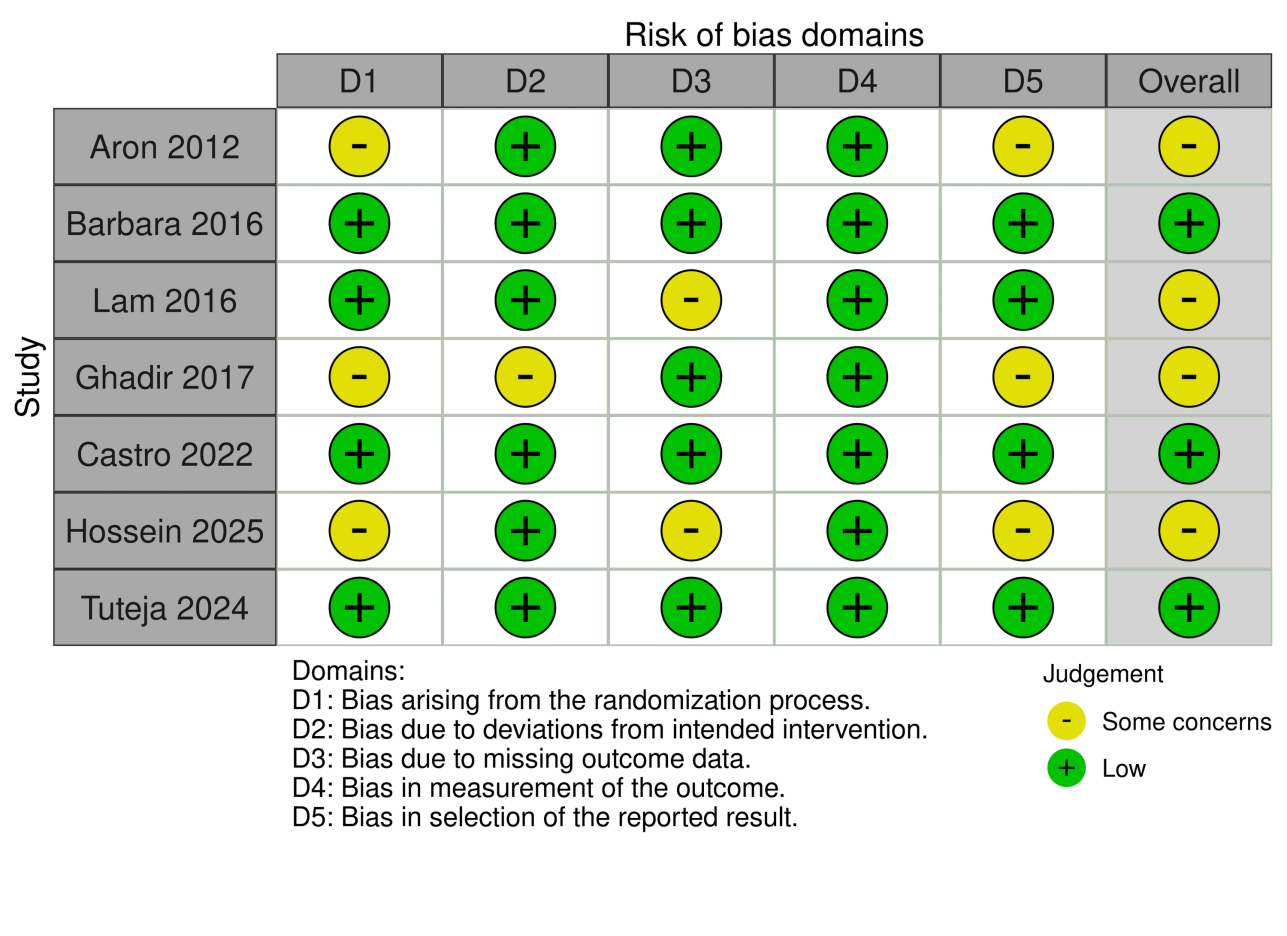
Risk of bias assessment of included studies using the Cochrane Risk of Bias 2 (RoB 2) tool. The individual risk of bias judgments for each included study. This figure (traffic light plot) summarizes the risk of bias (RoB) assessments in each domain of the Cochrane RoB 2 tool across all seven included studies [22-28] RoB 2 tool: risk of bias 2 tool; D1: bias arising from the randomization process; D2: bias due to deviations from intended interventions; D3: bias due to missing outcome data; D4: bias in measurement of the outcome; D5: bias in selection of the reported result.

**Figure 3:**
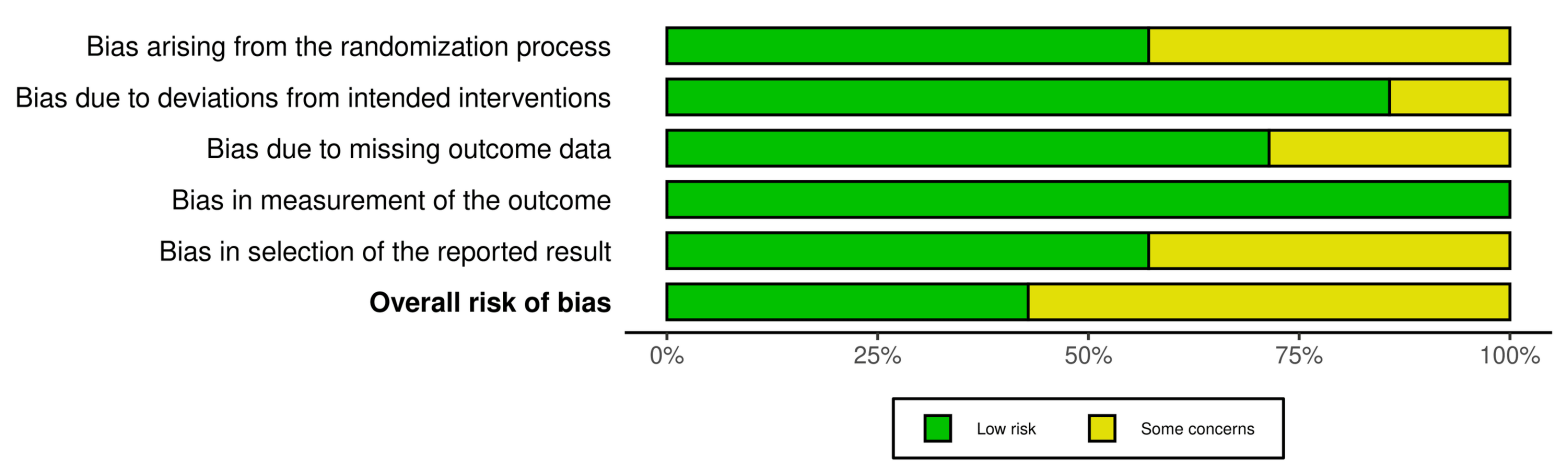
The proportion of studies classified as low risk (green) or some concerns (yellow) across each bias domain. The studies included in this figure correspond to references [22-28].

Overall, the included studies showed low to moderate risk of bias. Most studies were judged as low risk in the domains of deviations from intended interventions, missing outcome data, and measurement of the outcome. However, some concerns were identified mainly in the domains of bias arising from the randomization process and bias in the selection of the reported results. The summary risk of bias graph and the individual study-level judgments are shown in ​​​​Figures 2, 3.

The summary risk of bias graph showed that concerns were most frequent in Domain 1 and Domain 5, whereas Domain 4 showed consistently low risk across studies. None of the included studies showed a high overall risk of bias.

Global Irritable Bowel Syndrome (IBS) Symptom Response

Four RCTs involving 569 participants were included in the meta-analysis of global IBS symptom response [22-25]. Pooled analysis using a random-effects model showed no statistically significant difference between mesalazine and placebo: RR = 1.11, 95% CI 0.93-1.32; I² = 10%; p = 0.27.

**Figure 4:**
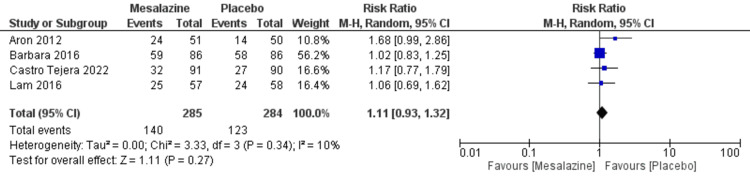
Forest plot of global IBS symptom response comparing mesalazine versus placebo using a random-effects model. Forest plot showing the effect of mesalazine versus placebo on global irritable bowel syndrome symptom response using risk ratio and a random-effects model. Values greater than one favor mesalazine, while values less than one favor placebo. The studies included correspond to references [22-25]. CI: confidence interval; M-H: Mantel-Haenszel; RR: risk ratio; df: degrees of freedom.

Heterogeneity was low, suggesting good consistency across studies. Overall, mesalazine did not significantly improve the proportion of patients achieving global symptom response compared with placebo (Figure 4).

Abdominal Pain

Three studies comprising 193 participants evaluated abdominal pain as a continuous outcome [26-28]. The pooled analysis showed no statistically significant effect of mesalazine over placebo: SMD = −0.10, 95% CI −0.82 to 0.61; I² = 83%; p = 0.77.

**Figure 5:**
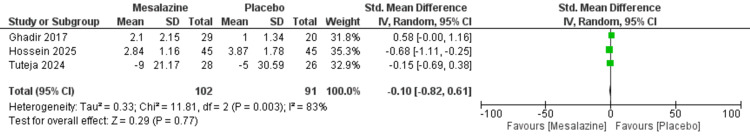
Forest plot of abdominal pain (continuous outcome) comparing mesalazine versus placebo using the standardized mean difference. Forest plot showing the effect of mesalazine versus placebo on abdominal pain using standardized mean difference and a random-effects model. Negative values favor mesalazine, while positive values favor placebo. The studies included correspond to references [26-28]. SD: standard deviation; CI: confidence interval; IV: inverse variance; SMD: standardized mean difference; df: degrees of freedom.

This analysis was characterized by substantial heterogeneity, indicating marked variability between studies. Differences in patient selection, IBS subtype, baseline symptom burden, treatment duration, and pain measurement scales may partly explain this heterogeneity (Figure 5).

Global Irritable Bowel Syndrome (IBS) Symptom Score

Three RCTs, including 325 participants, evaluated global IBS symptom score as a continuous outcome [24,27,28]. The pooled analysis demonstrated a statistically significant improvement in favor of mesalazine: SMD = −0.39, 95% CI −0.64 to −0.15; I² = 16%; p = 0.002. The observed effect size for global IBS symptom score corresponds to a small to moderate clinical effect.

**Figure 6:**
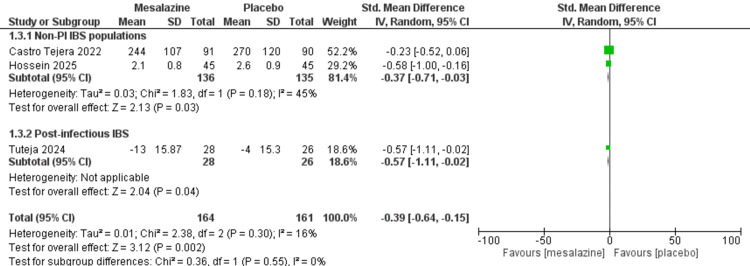
Forest plot of Global IBS Symptom Score (continuous outcome) comparing mesalazine versus placebo using standardized mean difference. Forest plot showing the effect of mesalazine versus placebo on global irritable bowel syndrome symptom score using standardized mean difference and a random-effects model, with subgroup analysis according to post-infectious status. Negative values favor mesalazine, while positive values favor placebo. The studies included correspond to references [24,27,28]. CI: confidence interval; df: degrees of freedom; IBS: irritable bowel syndrome; IV: inverse variance; PI-IBS: post-infectious irritable bowel syndrome; SD: standard deviation; SMD: standardized mean difference.

Heterogeneity was low, supporting the consistency of this finding. Subgroup analysis according to IBS phenotype showed improvement in both non-post-infectious IBS and post-infectious IBS, with no significant subgroup differences (p = 0.55). These findings suggest that although mesalazine did not significantly improve dichotomous global response or abdominal pain, it may reduce overall IBS symptom burden when assessed using continuous symptom scales (Figure 6).

Publication Bias

Because only seven RCTs were included overall, and fewer than 10 studies contributed to each pooled outcome, formal assessment of publication bias using funnel plots or statistical asymmetry tests was not considered reliable. Therefore, publication bias was assessed qualitatively.

Although no definitive conclusion regarding publication bias can be drawn, the relatively small number of included studies and the modest sample sizes of several trials warrant cautious interpretation of the pooled results.

Certainty of Evidence (Grading of Recommendations, Assessment, Development and Evaluation (GRADE))

The certainty of the evidence represents the degree of confidence that the observed effect estimate approximates the true effect. The certainty of evidence for each outcome was evaluated using the Grading of Recommendations, Assessment, Development and Evaluation (GRADE) framework.

For global IBS symptom response, the certainty of evidence was rated as moderate. Although statistical heterogeneity was low (I² = 10%), the certainty was downgraded due to concerns about risk of bias, particularly in the domains of randomization and selective reporting.

For abdominal pain, the certainty of evidence was considered low. This rating was mainly attributable to substantial between-study heterogeneity (I² = 83%) and imprecision, as reflected by wide confidence intervals that crossed the line of no effect.

For the global IBS symptom score, the certainty of evidence was rated as moderate. Despite low heterogeneity (I² = 16%) and statistically significant findings, the certainty was downgraded due to concerns about the risk of bias across the included studies. Overall, these certainty ratings indicate that future studies could have an important impact on the estimated effects, especially for abdominal pain, where heterogeneity and imprecision reduce confidence in the findings. A summary of the findings table is presented in Table 2.

**Table 2:** Summary of findings for mesalazine compared with placebo in patients with irritable bowel syndrome. The studies included correspond to references [22-28]. RR: risk ratio; SMD: standardized mean difference; CI: confidence interval; IBS: irritable bowel syndrome; GRADE: Grading of Recommendations, Assessment, Development and Evaluation; ⨁⨁⨁◯: moderate certainty of evidence; ⨁⨁◯◯: low certainty of evidence. GRADE Working Group grades of evidence: High certainty: Further research is very unlikely to change our confidence in the estimate of effect; Moderate certainty: Further research is likely to have an important impact on our confidence in the estimate and may change the estimate; Low certainty: Further research is very likely to have an important impact on our confidence in the estimate and is likely to change the estimate; Very low certainty: Any estimate of effect is very uncertain. Downgrading decisions: Global IBS symptom response was downgraded due to some concerns in risk of bias across included studies; Abdominal pain was downgraded due to substantial heterogeneity (I² > 75%) and imprecision (wide confidence intervals); Global IBS symptom score was downgraded due to some concerns in risk of bias.

Outcome	No. of studies (participants)	Effect estimate (95% CI)	Heterogeneity (I²)	Certainty of evidence (GRADE)	Interpretation
Global IBS symptom response	4 RCTs (n = 569)	RR 1.11 (0.93 to 1.32)	10%	⨁⨁⨁◯ Moderate	No significant difference between mesalazine and placebo
Abdominal pain (continuous)	3 RCTs (n = 193)	SMD −0.10 (−0.82 to 0.61)	83%	⨁⨁◯◯ Low	No significant effect; substantial heterogeneity observed
Global IBS symptom score	3 RCTs (n = 325)	SMD −0.39 (−0.64 to −0.15)	16%	⨁⨁⨁◯ Moderate	Significant improvement favoring mesalazine

Discussion

Principal Findings

In this systematic review and meta-analysis of randomized clinical trials, mesalazine did not significantly improve global IBS symptom response or abdominal pain compared with placebo, although a modest but statistically significant improvement was observed in global IBS symptom scores. These findings highlight the complex and heterogeneous nature of IBS and suggest that the potential therapeutic role of mesalazine may be limited to specific outcomes or patient subgroups rather than providing a consistent clinical benefit across all domains.

These findings are consistent with a recent meta-analysis by Goodoory et al. [29], which also reported no significant benefit of mesalazine in IBS, reinforcing the limited role of anti-inflammatory therapy in unselected populations.

Interpretation of Efficacy Outcomes

The lack of effect on global symptom response is particularly relevant, as this outcome is considered the most clinically meaningful endpoint in IBS trials and is widely used in regulatory and guideline-based assessments [4,5,17,18]. The pooled estimate showed a neutral effect with low heterogeneity, indicating consistent findings across studies.

IBS trials are characterized by substantial placebo response rates, which may reduce the apparent treatment effect of pharmacological interventions and complicate the interpretation of efficacy outcomes. This may partly explain why mesalazine did not significantly improve dichotomous global symptom response despite the modest improvement observed in continuous global IBS symptom scores.

Similarly, mesalazine did not significantly reduce abdominal pain, one of the hallmark symptoms of IBS. Abdominal pain in IBS is multifactorial and driven by visceral hypersensitivity, dysregulation of the gut-brain axis, and central pain processing mechanisms [6-8,30], which are unlikely to be adequately targeted by anti-inflammatory therapy alone. The substantial heterogeneity observed in the abdominal pain analysis may reflect differences in baseline pain severity, IBS subtype distribution, pain assessment instruments, treatment duration, and the multifactorial mechanisms underlying pain generation in IBS.

In contrast, mesalazine was associated with a modest improvement in global IBS symptom scores when analyzed as a continuous outcome. This discrepancy between dichotomous and continuous outcomes has been described in IBS trials and may reflect the higher sensitivity of continuous scales to detect subtle changes in symptom burden [17,18]. However, although this finding was statistically significant, the magnitude of the pooled standardized mean difference was modest, and its clinical relevance remains uncertain. Continuous symptom scales may detect small changes in symptom burden that do not necessarily translate into clinically meaningful responder outcomes.

Pathophysiological and Clinical Implications

From a pathophysiological perspective, these findings align with the current understanding of IBS as a heterogeneous disorder in which low-grade inflammation represents only one of multiple contributing mechanisms [9-11,31]. While immune activation has been demonstrated in subsets of patients, particularly those with post-infectious IBS [12-14], its role is variable and not universally present across all IBS phenotypes.

Importantly, previous studies have suggested that anti-inflammatory strategies may be more effective in selected subgroups of IBS patients with objective evidence of mucosal immune activation or increased intestinal permeability [31,32]. However, the included trials largely enrolled unselected IBS populations, which may dilute potential treatment effects. Clinical heterogeneity among IBS subtypes may also influence treatment response. IBS with diarrhea, IBS with constipation, mixed IBS, and post-infectious IBS differ in symptom profile, underlying pathophysiological mechanisms, and degree of immune activation. Since mesalazine primarily targets mucosal inflammation, its potential benefit may be greater in selected patients with post-infectious IBS or objective evidence of low-grade inflammatory activity rather than in unselected IBS populations.

Subgroup analyses according to IBS subtype, post-infectious IBS status, or inflammatory biomarkers would have been clinically informative, particularly given the proposed anti-inflammatory mechanism of mesalazine. However, these analyses were not feasible because of the limited number of trials and inconsistent reporting of these variables across studies.

Methodological Considerations

The overall methodological quality of the included studies was acceptable, although domain-specific concerns were identified, particularly regarding the randomization process and selective reporting. Additionally, sample sizes were generally modest, and outcome definitions varied across studies.

Limitations

The findings should be interpreted in light of several limitations. First, the relatively small number of included studies may limit statistical power, particularly for subgroup analyses. Sensitivity analyses were planned to assess the robustness of the pooled estimates; however, they could not be performed because of the limited number of eligible trials and the small number of studies contributing data to each outcome. Therefore, the stability of the pooled findings should be interpreted with caution.

Second, substantial clinical heterogeneity was observed, especially in the abdominal pain analysis. This may reflect differences in IBS subtype, symptom severity, treatment duration, outcome measurement, and patient selection across trials.

Third, the use of standardized mean differences may limit clinical interpretability because the pooled estimate reflects relative differences across different symptom scales rather than a direct change in a single clinically familiar unit.

Finally, formal assessment of publication bias using funnel plots was not feasible because fewer than 10 studies were included in each pooled analysis, limiting the reliability of visual asymmetry assessment.

Clinical Relevance and Future Directions

Despite these limitations, our findings are consistent with current clinical guidelines, which do not recommend mesalazine for routine IBS management [4,5,17,18]. From a clinical perspective, these findings do not support the routine use of mesalazine in unselected IBS populations and suggest that its use should be limited to selected patients with evidence of mucosal immune activation or post-infectious IBS. These results reinforce the need for a personalized, mechanism-based therapeutic approach rather than empiric treatment strategies.

Beyond symptom control, future studies should explore whether the anti-inflammatory effects of mesalazine may have disease-modifying implications in selected IBS phenotypes with mucosal immune activation. Although 5-ASA has been associated with reduced colorectal neoplasia risk in inflammatory bowel disease [33,34], IBS is not considered a premalignant inflammatory condition. Therefore, any potential chemopreventive role of mesalazine in IBS remains speculative and would require long-term longitudinal studies.

## Conclusions

In conclusion, mesalazine did not significantly improve global IBS symptom response or abdominal pain in patients with irritable bowel syndrome. Although a modest statistically significant improvement was observed in overall IBS symptom scores when analyzed as a continuous outcome, the clinical relevance of this finding remains uncertain. Current evidence does not support the routine use of mesalazine in unselected IBS populations.

The possibility that selected IBS phenotypes, such as patients with post-infectious IBS or objective evidence of mucosal immune activation, may derive benefit from anti-inflammatory therapy remains hypothesis-generating and cannot be confirmed based on the available evidence. Future adequately powered randomized controlled trials should incorporate biomarker-driven patient selection, standardized and clinically meaningful outcome measures, predefined subgroup analyses, and longer follow-up periods. Overall, these findings support further investigation of a precision medicine approach in IBS, in which treatment selection is guided by underlying pathophysiological mechanisms rather than empiric therapy in unselected populations.

## Appendices

Appendix 1 

**Table 3:** Database-specific search strategies used in the systematic review. Searches were conducted from database inception to March 23, 2026. Google Scholar results were screened by relevance, limited to the first 100 records. No language restrictions were applied during the initial search. IBS: irritable bowel syndrome; 5-ASA: 5-aminosalicylic acid.

Database/source	Search strategy	Filters/limits	Date searched
PubMed	(mesalazine OR mesalamine OR 5-ASA) AND (irritable bowel syndrome OR IBS) AND (randomized)	No language restrictions	March 23, 2026
ScienceDirect	(mesalazine OR mesalamine OR 5-ASA) AND (irritable bowel syndrome OR IBS) AND randomized	Original research articles	March 23, 2026
Google Scholar	(mesalazine OR mesalamine OR 5-ASA) AND (irritable bowel syndrome OR IBS) AND randomized	First 100 records sorted by relevance	March 23, 2026
Manual search	Reference lists of included studies	Additional relevant randomized trials	March 23, 2026
